# Combination of laparoscope and choledochoscope to treat biliary ascariasis: A CARE-compliant case report

**DOI:** 10.1097/MD.0000000000006291

**Published:** 2017-03-31

**Authors:** Ming Cai, Ji Cheng, Wei Li, Xiaoming Shuai, Jinbo Gao, Kailin Cai, Jiliang Wang, Jie Bai, Colin Rog, Guobin Wang, Kaixiong Tao

**Affiliations:** a Department of Gastrointestinal Surgery, Union Hospital, Tongji Medical College, Huazhong University of Science and Technology, Wuhan, China; b Baylor College of Medicine, Huston, TX.

**Keywords:** biliary ascariasis, case report, choledochoscope, laparoscope

## Abstract

**Rationale:**

Ascariasis is an endemic parasitic disease caused by Ascaris lumbricoides, which severely burdens the healthcare system as well as harms the personal life quality, especially among less developed regions. Biliary ascariasis is a critical complication of intestinal ascariasis with painful and life-threatening manifestations. The exploration of proper strategies as its medical interventions remains largely controversial.

**Patient concerns:**

A 16 year-old patient complained of abdominal pain and yellow sclera.

**Diagnoses:**

Biliary ascariasis

**Interventions:**

Laparoscopic cholecystectomy and bile duct exploration.

**Outcomes:**

More than one hundred ascarids were extracted and the patient had been discharged from hospital without any complications.

**Lessons:**

The combination of laparoscope and choledochoscope is an efficient method to treat biliary ascariasis, despite of large worm burden in the common bile duct.

## 1 Introduction

Ascariasis is an endemic parasitic disease caused by *Ascaris lumbricoides*, which severely burdens the healthcare system as well as harms the personal life quality, especially among less developed regions. Biliary ascariasis is a critical complication of intestinal ascariasis with painful and life-threatening manifestations. The exploration of proper strategies as its medical interventions remains largely controversial.

## 2 Case report

A 16-year-old female suffered from an abdominal pain at right upper quadrant, lasting for more than 10 days. Shortly ahead of her medical consultation, 4 ascaris-like worms were vomited out, individually with a length of about 10 cm. She had a previous onset of ascariasis, during which low-grade fever (38.4°C) occurred without apparent jaundice, diarrhea, or anemia. Physical examination revealed tenderness at right upper quadrant. Her total leukocyte count was 11.2 G/L consisting of 5.2% eosinophils. Serum and urine amylase were 386 and 928 U/L, respectively. In terms of liver functionality, the level of total bilirubin rose up to 23.2 μm/L, meanwhile the hepatic enzymes were similarly elevated (alanine aminotransferase 163 U/L; aspartate aminotransferase 96 U/L). Abdominal ultrasound described the enlargement of the gallbladder, upper segment of common bile duct (1.5 cm in diameter) and intrahepatic bile duct (1.3 cm in diameter). Furthermore, the intrahepatic bile duct was also discovered to be filled with echogenic and banded substances. Based on such evidences, she was clinically diagnosed as biliary ascariasis with a concomitant manifestation of biliary duct infection.

## 3 Methods and explanation

**Figure 1:**
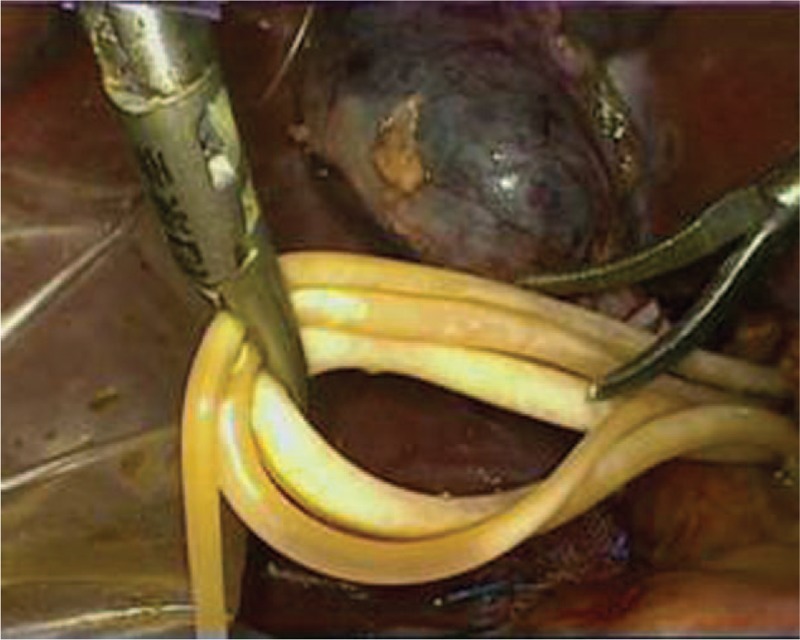
After laparoscopic choledochotomy, worms were extracted from the common bile duct.

**Figure 2:**
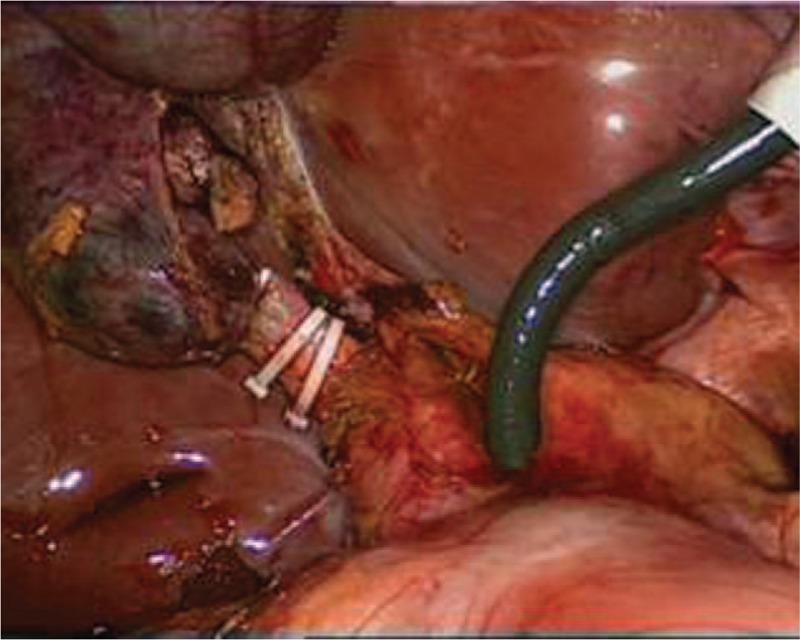
Laparoscopic choledochoscopy was performed to find hidden worms.

**Figure 3:**
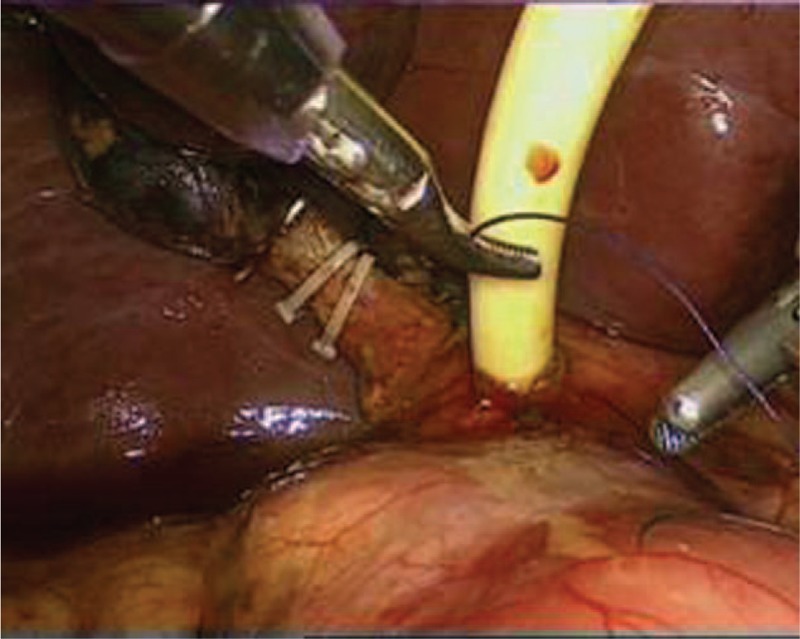
T-tube was placed in the common bile duct under laparoscope.

**Figure 4:**
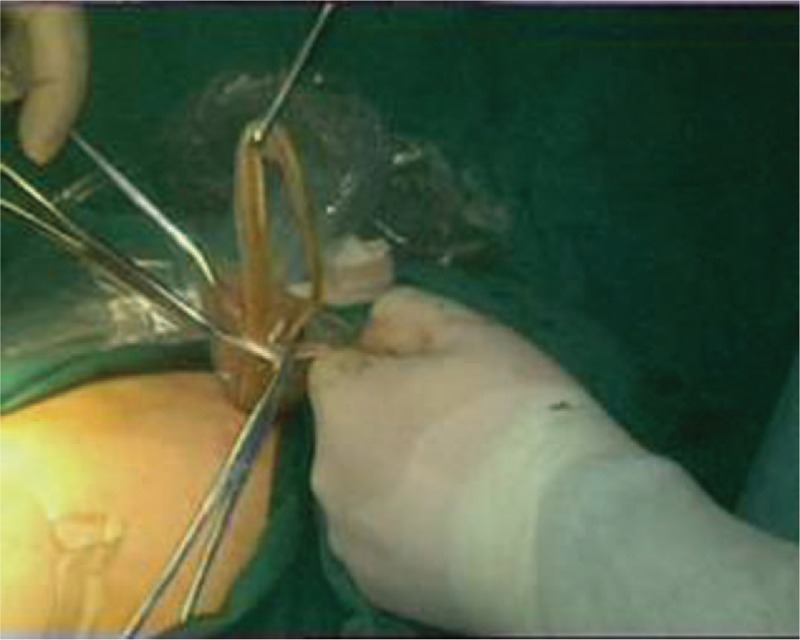
Plastic bag filled with worms was removed from the abdominal cavity via trocar port.

**Figure 5:**
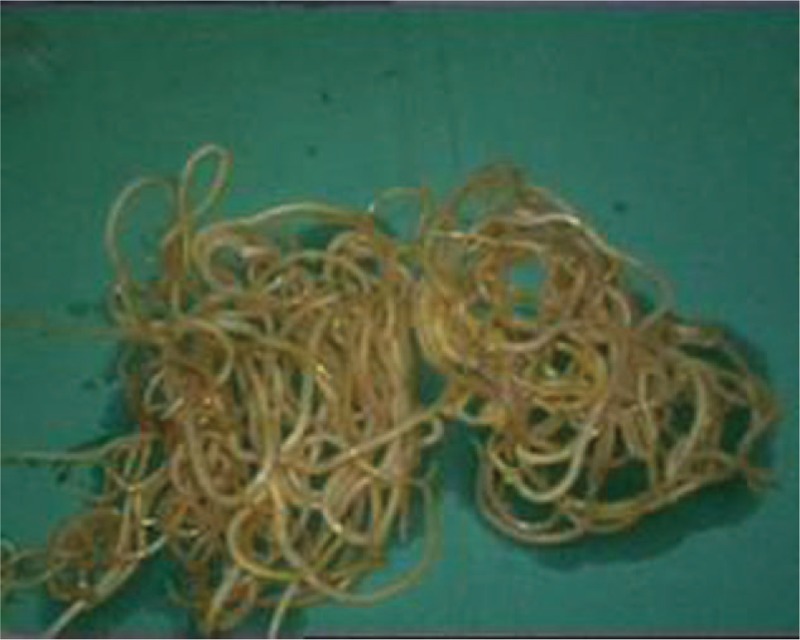
About 100 worms were extracted from the common bile duct.

Through a laparoscopic exploration, the enlarged gallbladder and common bile duct were visually confirmed, as well as the massive storage of worms within the biliary tract. Subsequently, a 1 cm incision was made on the common bile duct in order to facilitate the extraction of the pathogenic worms, which were then placed into a specimen bag laparoscopically (Fig. 1). Once the contained worms were virtually cleared out under laparoscope, chodedochoscopy was employed to seek for more hidden worms inside the biliary system (Fig. 2). After finishing the removal of ascarides, the biliary duct incision was well sutured, with a T-tube placed in situ (Fig. 3). Eventually, a standard laparoscopic cholecystectomy was performed, followed by the removal of specimen bags and drainage tube placement (Fig. 4). The total amount of worms inside the common bile duct summed up to more than 100 (Fig. 5). Postoperatively, she was treated with oral antihelminthic drugs for 3 days. The T-tube was rinsed with saline every day, and during the first 3 days, 6 more worms were additionally washed out. The patient experienced a smooth postoperative recovery and was finally discharged from hospital without any complications.

## 4 Discussion

Ascariasis is an endemic parasitic disease that mostly prevails in developing countries with lower standards of public health and hygiene, such as Southeast Asia, Central Africa, and South America. Currently, it affects more than 1 billion people worldwide, however, mainly among juvenile population.^[1]^

Usually, *A lumbricoides* remains in the small intestine without causing any significant annoyance. However, when the environment in the digestive tract becomes unsuitable, the parasite adaptively migrates to alternate locations especially toward narrow lumens. From the duodenum, the adult worms are able to pass through the major duodenal papilla and toward the hepatobiliary system, leading to biliary ascariasis. Infestation of the bile duct can cause biliary colic, acute cholangitis, acute cholecystitis, acute pancreatitis, and, rarely, hepatic abscesses.^[2,3]^ Furthermore, residuum of dead worms can serve as a nidus for hepatic stone formation in the common bile duct, and living worms release glucuronidase which can deconjugate bilirubin and lead to its precipitation and formation into pigment stones.

Standard treatment of biliary ascariasis includes antihelminthic drug therapy and either endoscopic or surgical extraction.^[4,5]^ Surgery plays a core role for patients who have failed antihelminthic treatment and endoscopic extraction.

## 5 Conclusion

The authors report a rare case of biliary ascariasis with more than 100 adult worms inhabiting the common bile duct. In order to extract as many worms as possible, the authors performed laparoscopic extraction and chodedochoscopic reexamination to secure the safety and efficacy. The authors also placed a T-tube in situ that could be rinsed daily with saline to flush out remaining parasites. From this case, the authors demonstrate that the combination of laparoscope and choledochoscope is an efficient method to treat biliary ascariasis with large worm burden in the common bile duct.

## Acknowledgments

The authors sincerely thank all staff in our department for offering proper assistance during the medical services and manuscript writing process.
